# Response to the letter to the editor from Morfeld

**DOI:** 10.1007/s00420-016-1121-y

**Published:** 2016-02-22

**Authors:** Matthias Möhner

**Affiliations:** Division of Work and Health, Federal Institute for Occupational Safety and Health, Nöldnerstr. 40/42, 10317 Berlin, Germany

I would like to thank the Journal for giving me the opportunity to respond to comments from Dr. Morfeld on my suggested approach to adjust standardized mortality ratios (SMRs) for competing causes.

The aim of my suggested approach (Möhner 2015) was to develop a method, which can even be applied to published results from cohort studies like the US coal miner mortality study (Attfield and Kuempel 2008), which I used as an example. Contrary to Dr. Morfeld’s assumption, neither the number of observed cases nor the overall number of expected cases has been changed by my proposed approach. This is reflected in the basic assumption ${\text{SMR}}^{*} = {\text{SMR}}$, using the notations of the primary paper. The idea of the approach is to merely change the partition of the expected cases in an appropriate manner, assuming that the mortality in the adjusted reference population is proportional to that in the original one with the exception that the mortality due to the competing cause is similar to the observed mortality in the cohort. The latter assumption can be rewritten as ${\text{SMR}}_{0}^{*} = 1$. Such a strong condition can, however, entail an overestimation of the risk estimators for single endpoints in case of a considerably elevated overall SMR. Therefore, the assumption has been modified in my approach to ${\text{SMR}}_{0}^{*} {\text{ = SMR}}$ to compensate for a possible overestimation.

In his comment, Dr. Morfeld presented an alternative method to adjust for competing causes leading to a SMR = 1.03 in the worked example. However, he has overseen that after blocking pneumoconiosis as a cause of death the probability of a certain cause can then be described by the ratio between the observed number of this cause and the number of all causes of death except for pneumoconiosis. Therefore, this approach yields SMR = (7 + 6.7)/10.14 = 1.35 for the worked example, which even exceeds the originally proposed adjusted risk estimate. Following Dr. Morfeld’s recommendation, 67*67/137 = 32.8 miners suffering from pneumoconiosis would survive forever or at least up to the end of follow-up. However, this is exactly what he was actually trying to avoid by his recommendation.

In his criticism, Dr. Morfeld refers to a time-dependent method for a counterfactual or potential outcome model (Robins 1998) and his own calculations based on a cohort of German coal miners (Morfeld and Lampert 2004; Morfeld et al. 2005) as a kind of gold standard. The counterfactual concept refers to what would have happened if, contrary to fact, the exposure had been something other than what it actually was. Applying this concept to our exemplary research question, we may analyze what would have happened if a worker had worked as a construction worker on surface, for example, instead of working underground as a coal miner as he did in reality. Of course, in that case he would have not been exposed to coal dust, but his smoking habits could have been quite different also. Smoking is strictly prohibited in underground coal mines, and all miners, purely for reasons of self-preservation, respect this prohibition strictly during the whole underground shift. Therefore, it is plausible that a coal miner smokes noticeably less than his counterfactual sibling, and hence, the potential increase in lung cancer risk due to the exposure to coal dust might be even more than compensated by his reduced tobacco consumption. In addition, breathing difficulties due to a reduced lung function or receiving advice from the occupational health physician who has already diagnosed an early stage of coal workers’ pneumoconiosis may impact the smoking habit of the miner.

Regarding potential outcome models, innovative statistical methods like g-estimation are able to estimate causal effects even for difficult relationships as between coal dust, lung function, pneumoconiosis, smoking, and lung cancer—at least in theory. Detailed data describing the temporary course of the exposure of interest, of confounding factors, and of intermediate variables must be available to practically implement these methods and to take into account feedback mechanisms between these variables. Moreover, these data must be available for exposed as well as for unexposed study subjects. Thus, the hurdles for the practical implementation in occupational epidemiology are very high or almost impossible to overcome as in the case of retrospective cohort studies when data on smoking are also required. The German coal miner cohort (Morfeld et al. 2005) is unfortunately no exception concerning this matter. It lacks an unexposed reference group and offers only incomplete and selective data on smoking.

Another constraint of this cohort is its selection bias. The average time of employment underground for the cohort was almost 24 years at study entry (Morfeld et al. 2002). Moreover, 28 % of cohort members were only enrolled into the study after cessation of work underground, whereby the time span between cessation of work underground and study entry may have been up to 12 years (Morfeld et al. 1997). This strong selection bias due to left truncation and left censoring cannot be compensated by even the most ingenious statistical method. It is well known that this type of bias leads generally to an underestimation of risks (Applebaum et al. 2007, 2011). Hence, g-estimation should be applied only if the cohort is an inception cohort, i.e., consists of incident hires only (Chevrier et al. 2012).

Finally, I would like to emphasize that my suggested approach is of course a crude approximation only and, hence, may overestimate as well as underestimate the real risk. Yet the unadjusted SMR generally leads to an underestimation of the underlying risk. The proposed approach leads to a noticeable increase in the SMR only if major proportion of deaths are attributed to the competing cause of death. In case of the German coalminer cohort, where only 2.5 % of deaths are attributed to pneumoconiosis (Morfeld et al. 2005), the approach results in an increase in the SMR for lung cancer from 0.889 to just 0.905 for the complete cohort, corresponding to a relative change of 1.8 %. Applying the adjustment only to the subcohort of coal miners suffering from pneumoconiosis, the SMR increases somewhat more strongly from 1.74 to 1.89 by 8.6 %.
